# Weight Change across Adulthood in Relation to Non-Alcoholic Fatty Liver Disease among Non-Obese Individuals

**DOI:** 10.3390/nu14102140

**Published:** 2022-05-20

**Authors:** Yuqing Ding, Xin Xu, Ting Tian, Chengxiao Yu, Xinyuan Ge, Jiaxin Gao, Jing Lu, Zijun Ge, Tao Jiang, Yue Jiang, Hongxia Ma, Ci Song, Zhibin Hu

**Affiliations:** 1 Department of Epidemiology, School of Public Health, Nanjing Medical University, Nanjing 211166, China; dyqdyq226@163.com (Y.D.); xuxin0061@163.com (X.X.); tianting0628@njmu.edu.cn (T.T.); yuchengxiao@njmu.edu.cn (C.Y.); gxy_iris499@163.com (X.G.); jiaxingao131@163.com (J.G.); lj_123456@126.com (J.L.); tao.chiang0923@njmu.edu.cn (T.J.); jiangyue@njmu.edu.cn (Y.J.); hongxiama@njmu.edu.cn (H.M.); 2 Department of Health Promotion Center, Jiangsu Province Hospital and the First Affiliated Hospital of Nanjing Medical University, Nanjing 210029, China; 3 Office of Infection Management, Jiangsu Province Hospital and the First Affiliated Hospital of Nanjing Medical University, Nanjing 210029, China; gzj0408@126.com; 4 Research Units of Cohort Study on Cardiovascular Diseases and Cancers, Chinese Academy of Medical Sciences, Beijing 100730, China

**Keywords:** weight gain, NAFLD, population attributable fraction

## Abstract

Background: To investigate the associations of weight change patterns across adulthood with the risk of non-alcoholic fatty liver disease (NAFLD). Methods: Using data from the National Health and Nutrition Examination Survey (NHANES) 2017–2018 cycle, we performed a retrospective cohort study with 2212 non-obese participants aged 36 years old over. Weight change patterns were categorized as “stable non-obese”, “early adulthood weight gain”, “middle and late adulthood weight gain” and “revert to non-obese” according to the body mass index (BMI) at age 25, 10 years prior and at baseline. Vibration-controlled transient elastography (VCTE) was performed to diagnose NAFLD. Modified Poisson regression was used to quantify the associations of weight change patterns with NAFLD. Results: Compared with participants in the “stable non-obese” group, those who gained weight at early or middle and late adulthood had an increased risk of NAFLD, with an adjusted rate ratio (RR) of 2.19 (95% CI 1.64–2.91) and 1.92 (95% CI 1.40–2.62), respectively. The risk of NAFLD in “revert to the non-obese” group showed no significant difference with the stable non-obese group. If the association of weight change and NAFLD was causal, we estimated that 73.09% (95% CI 55.62–82.93%) of incident NAFLD would be prevented if the total population had a normal BMI across adulthood. Conclusions: Weight gain to obese at early or middle and late adulthood was associated with an evaluated risk of NAFLD. A large proportion would have been prevented with effective weight intervention.

## 1. Introduction

Non-alcoholic fatty liver disease (NAFLD) is the most prevalent form of liver disease, affecting nearly one billion adults worldwide [1,2,3]. This multisystem condition is associated with an increased risk of liver-related and cardiovascular extrahepatic diseases [4,5]. Therefore, NAFLD prevention of a high-risk population is the cornerstone of lowering the occurrence of related morbidity and mortality.

Obesity is an established risk factor for NAFLD [6]; nearly 80% of obese adults will progress to NAFLD [7]. Despite this, around 10–40% of NAFLD occurs in those with a normal body mass index (BMI), often called non-obese NAFLD [8,9]. A series of risk factors has been described related to non-obese NAFLD, such as visceral obesity, high fructose intake, cholesterol intake, and genetic risk factors (e.g., palatin-like phospholipase domain-containing 3) [10]. Longitudinal studies found that short-term weight gain remained an independent risk factor for NAFLD development in non-obese individuals [11,12]. By contrast, tracking long-term weight may accurately and integrally reflect the dynamic change of excess body fat through an entire life, thereby improving the proposal of health guidance or recommendation on weight control for different age phases. Weight gain through early and middle adulthood may be relevant to NAFLD because of growth of age and excess adiposity deposition during this period [13,14].

To date, few studies have solely investigated the impact of early and midlife adulthood weight gain on NAFLD risk. A recent study based on the Nurses’ Health Study II cohort found that both early and lifetime adulthood weight gain trajectories were independently associated with an excess risk of developing NAFLD [15]. Although based on a well-designed cohort, the generalizability of the findings was constrained by the fact that the samples were female and not nationally representative (selection bias), and NAFLD ascertainment was self-reported (information bias). The National Health and Nutrition Examination Survey (NHANES) is a nationally representative designed survey. The questionnaire contains weight history questions, including recalled weight and height at age 25 and recalled weight at 10 years prior to baseline, alongside measured weight and height at the baseline survey. NAFLD It also diagnosed by using an accurate and quantified application of vibration-controlled transient elastography (VCTE). Taking advantage of the NHANES 2017–2018 database, we have an opportunity to investigate the association between histories of weight gain and the incidence of NAFLD.

Thus, using the nationally representative NHANES 2017–2018 data, we aimed to (1) determine whether non-obese individuals who gained weight during early and middle adulthood were at an increased risk of NAFLD (the risk raise hypothesis); (2) determine whether non-obese individuals who converted from obese to non-obese through adulthood were at a reduced risk of NAFLD (the risk elimination hypothesis); (3) qualify the amount of NAFLD that would be prevented if the total population maintained a normal BMI during a life course.

## 2. Materials and Methods

### 2.1. Study Population

We used data from the 2017–2018 cycle of NHANES, which exclusively assessed hepatic steatosis and liver fibrosis by VCTE. The NHANES is a cross-sectional survey program well represented for the civilian United States population with a complex stratified, multistage and clustered probability sampling design [16,17,18]. We used recalled questions on weight history and NAFLD diagnosed by VCTE at baseline (after excluding self-reported fatty liver) to establish a retrospective cohort based on the cross-sectional data. The survey was approved by the National Center for Health Statistics Research Ethics Review Board (protocol number: 2018-01), and written informed consent was obtained from all participants.

In this study, participants aged ≥ 36 years old (*n* = 4218) at baseline were enrolled. Excluded from the study were pregnant women (*n* = 11), those who did not receive physical examination (*n* = 227), with BMI ≥ 30 kg/m^2^ at age 25 or missing BMI at age 25 or baseline (*n* = 287), and those who partially completed, were ineligible, or did not perform VCTE (*n* = 458). Then, we excluded participants with excessive alcohol consumption (≥2 drinks/day in men or ≥1 drink/day in women) (*n* = 150), exposure to hepatitis virus infection (*n* = 132), self-reported fatty liver and other liver conditions (*n* = 75), and self-reported malignancy (*n* = 366). In total, 2212 participants who were non-obese at age 25 were included in the analysis (Figure S1).

### 2.2. Measurement of Exposure: Weight Change Patterns

Baseline weight and height were measured during physical examination. Weight at age 25 and 10 years prior to baseline were recalled at in-person interview. BMI was calculated as weight (kg) divided by the square of height (m^2^). BMI at age 25 (BMI_age 25_) was calculated using recalled height and weight, considering the possibility of height declined with age. BMI at 10 years prior to baseline (BMI_10 prior_, mean age 45.74 years old) was calculated using recalled weight and measured height. BMI at baseline (BMI_baseline_, mean age 55.74 years old) was calculated using measured height and weight.

**Figure 1:**
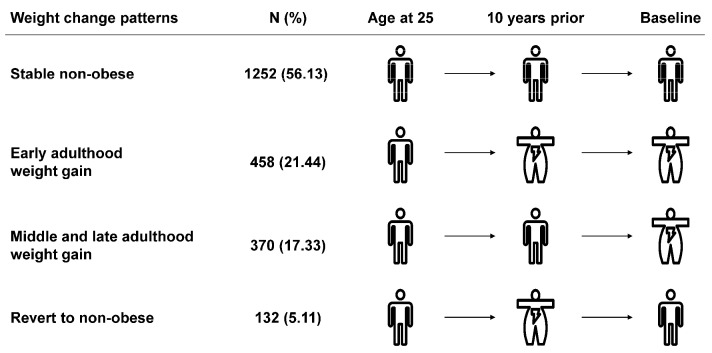
The definition of weight change patterns.

BMI change patterns were generated according to BMI at the three timepoints: “stable non-obese” (BMI_age 25_ < 30 kg/m^2^, BMI_10 prior_ < 30 kg/m^2^ and BMI_baseline_ < 30 kg/m^2^), “early adulthood weight gain” (BMI_age 25_ < 30 kg/m^2^, BMI_10 prior_ ≥ 30 kg/m^2^ and BMI_baseline_ ≥ 30 kg/m^2^), “middle and late adulthood weight gain” (BMI_age 25_ < 30 kg/m^2^, BMI_10 prior_ < 30 kg/m^2^ and BMI_baseline_ ≥ 30 kg/m^2^), “revert to non-obese” (BMI_age 25_ < 30 kg/m^2^, BMI_10 prior_ ≥ 30 kg/m^2^ and BMI_baseline_ < 30 kg/m^2^) (Figure 1).

### 2.3. Measurement of Outcome: Fatty Liver

In the 2017–2018 cycle, FibroScan^®^ model 502 V2 Touch (Echosens^TM^, Nevada, North America) was performed, equipped with a medium (M) (74% of participants) or extra-large (XL) probe. Controlled attenuation parameter (CAP) scores were derived as the indicator of hepatic steatosis, and liver stiffness measurement (LSM) scores were measured to detect liver fibrosis.

One was defined as NAFLD if he/she carried a median CAP score ≥ 280 dB/m (S3, severe fatty liver, the cutoff of sensitivity fixed at 82% [19]). Participants with a median LSM value of 9.7 kPa or more were considered with advanced fibrosis [20].

### 2.4. Covariates

Information on covariates was obtained through baseline questionnaires, including age (years), sex (male and female), race and ethnicity (non-Hispanic White, non-Hispanic Black, non-Hispanic Asian, Hispanic and other), education (less than high school, high school or equivalent, college or above), family income–poverty ratio level (0–1.0, 1.1–3.0, >3.0) and marital status (married, separated, never married). Alcohol consumption was defined as a non-drinker and low to moderate drinker (<2 drinks/day in men and <1 drink/day in women). Smoking status was grouped into never smoker, former smoker and current smoker. We defined leisure time physical activity level as 0 times/week, 1–2 times/week and ≥3 times/week. Healthy eating index scores (HEI-2015) were calculated to represent dietary quality. Diabetes was defined as fasting plasma glucose ≥ 126 mg/dL, HbA1c ≥ 6.5%, and/or currently taking insulin or diabetic pills [21]. Hypertension was defined as average blood pressure ≥ 140/90 mmHg, and/or receipt of an anti-hypertensive medication [22]. Dyslipidemia was defined as total cholesterol ≥ 240 mg/dL, and/or taking prescription for cholesterol [23].

### 2.5. Statistical Analysis

#### 2.5.1. Baseline Description

Appropriate sampling weights, clusters, and stratums were applied to this study. Continuous variables were presented as weighted mean ± standard errors (SE), and categorical variables were presented as numbers with percentages. We compared baseline characteristics by weight change patterns using linear regression adjusted for sampling weights for continuous variables and Rao-Scott χ^2^ test for categorical variables.

#### 2.5.2. Association Analysis

We qualified the association of weight change patterns with NAFLD using the multivariable modified Poisson regression model with a robust variance estimator to estimate rate ratios (RRs) and 95% confidence intervals (CI) directly, recognizing that odds ratios (ORs) may overestimate effect differences and do not give a good approximation of the RRs when the outcome is common (affecting > 10% of population). We adjusted baseline age, sex and race/ethnicity in model 1. Model 2 was additionally adjusted for education level, family income–poverty ratio level, marital status, alcohol consumption, smoking status, and chronic diseases. Model 3 was further adjusted for leisure time physical activity level and HEI. We also assessed the association between weight change patterns and advanced fibrosis in the secondary analysis.

#### 2.5.3. Calculation of Population Attributable Fraction (PAF)

We calculated PAF to estimate the percentage of NAFLD cases that could be prevented under the four hypothetical scenarios: (1) “weight loss”, defined as the scenario in which individuals who gained weight from early adulthood could have lost weight in later adulthood (“early adulthood weight gain” group vs. “revert to non-obese” group); (2) “weight maintenance”, defined as the scenario in which individuals who gained weight to obese could have maintained non-obese during adulthood (“early or middle and late adulthood weight gain” group vs. “stable non-obese” group); (3) “partial prevention”, defined as the scenario in which the total population maintained non-obese across adulthood (BMI < 30 kg/m^2^, “stable non-obese” vs. other counterparts); and (4) “comprehensive prevention”, defined as the scenario in which the total population had a normal BMI during the life course (BMI < 25 kg/m^2^, “stable normal” vs. other counterparts).

Consistent with prior studies [24], PAFs were calculated according to the following equation: $PAF=\sum_{i}pd_{i}\left(\frac{RR_{i}-1}{RR_{i}}\right)$, where *pd_i_* is the proportion of total NAFLD cases observed in the *i*th weight change pattern category and *RR_i_* is the adjusted rate ratio for the *i*th exposure category. A category-specific attributable fraction is the percentage of NAFLD cases that would be eliminated if individuals in that weight change category were to be shifted to another lower risk category, assuming a causal relation.

#### 2.5.4. Sensitivity Analysis

We performed a series of sensitivity analyses to access the stability of the results. First, we defined NAFLD as a median CAP value of 248 dB/m or more (≥S1, mild fatty liver) and a median CAP value of 268 dB/m or more (≥S2, moderate fatty liver). Second, we removed participants with baseline diabetes, hypertension and dyslipidemia to avoid inverse association. Third, we classified absolute weight change into five groups (weight loss of at least 2.5 kg, weight change within 2.5 kg, weight gain of at least 2.5 kg but less than 10.0 kg, weight gain of at least 10 kg but less than 20.0 kg and weight gain of at least 20.0 kg) and examined the association between absolute weight change group and NAFLD. Finally, the association between absolute weight change (continuous variable) and NAFLD was executed to explore the possible non-linear relation by using multivariable linear regression models based on restricted cubic splines with 4 knots.

All analyses were performed by Stata 16.0 (StataCorp, College Station, TX, USA), using Taylor series linearization. A two-tailed *p* value less than 0.05 was considered statistically significant.

## 3. Results

### 3.1. Baseline Characteristics

The correlation coefficients between BMI_age 25_, BMI_10 prior_, and BMI_baseline_ ranged from 0.397 to 0.668 (Table S1). The mean age was 55.74 years old at baseline and 46.22% were male. Throughout the whole adulthood, 1252 (56.13%) participants remained non-obese across adulthood (classified as “stable non-obese” group), 458 (21.44%) became obese from early adulthood (classified as “early adulthood weight gain” group), and 370 (17.33%) moved to obesity from middle and late adulthood (classified as “middle and late adulthood weight gain” group). There were also 132 (5.11%) participants who moved to obese at 10 years prior but reverted to the non-obese at baseline (classified as “revert to non-obese” group) (Figure 1).

Table 1 shows baseline characteristics of study participants. The “early adulthood weight gain” group had a higher proportion of non-Hispanic White, and the “middle and late adulthood weight gain” group had a higher proportion of non-Hispanic Black. Compared with the “stable non-obese” group, the weight gaining groups were more likely to be low to moderate drinkers, current smokers, physically inactive and have a lower healthy eating index score. Participants in the “revert to the non-obese” group were more likely to be former smokers, physically active and have healthier eating habit.

### 3.2. Relations of Weight Change Patterns with NAFLD

When evaluating the weight status at each time point (Table S2), we found that overweight and obesity were significantly associated with increased risks of NAFLD (*p* for trend < 0.001). Substantially rising curves were observed when we assessed the association between absolute weight changes and CAP value during the three-time intervals (Figure S2A). When describing the distribution of CAP value across the weight change patterns (Figure S2B), the “early adulthood weight gain” group had the highest median CAP value, the “middle and late adulthood weight gain” group came a close second, whereas the “revert to non-obese” group sharply declined, and the “stable non-obese” group came lowest (*p* < 0.001).

The association of weight change patterns across adulthood with NAFLD was presents in Table 2. The age-adjusted incidence rate of NAFLD among the four groups from the highest to the lowest is below: 67.20% (95% CI 58.75–75.64%) in the “early adulthood weight gain” group, 54.57% (95% CI 46.82–62.31%) in the “middle and late adulthood weight gain” group, 37.40% (95% CI 27.85–46.95%) in the “revert to the non-obese” group and 23.82% (95% CI 19.66–27.98%) in the “stable non-obese” group. Compared with the stable non-obese participants, those who gained weight in early adulthood were associated with a 119% higher risk of NAFLD (RR 2.19, 95% CI 1.64–2.91) and those who gained weight at middle and late adulthood had 92% higher risk of NAFLD (RR 1.92, 95% CI 1.40–2.62). Notably, the “revert to non-obese” group showed a null association with NAFLD, with RR of 1.01 (95% CI 0.62–1.64).

Regarding the risk raise hypothesis, participants who gained weight through adulthood had a 107% higher risk of NAFLD (RR 2.07, 95% CI 1.60–2.67) compared with those stable non-obese participants (Table 3). In the risk elimination hypothesis, we observed that the participants who reverted to non-obese had a 52% lower risk of NAFLD (RR 0.48, 95% CI 0.37–0.61) when compared with those who gained weight at early adulthood.

In the stratified analysis, we found that the associations were stronger among participants who were younger (*p* for interaction = 0.015, Figure S3A). The overall patterns were broadly comparable when stratified by sex (Figure S3B). We observed that the effects of weight gain were stronger in non-Hispanic Black when stratified by race and ethnicity; however, there were no significant interactions with race (*p* for interaction = 0.844, Figure S4).

In the secondary analysis, we reported the association of weight change patterns with advanced fibrosis in Table S3. Similarly, compared with the “stable non-obese” participants, those who gained at early adulthood or middle and late adulthood had a higher incidence risk of advanced fibrosis, with an RR of 3.20 (95% CI 1.19–8.62) and 2.46 (95% CI 0.97–6.20). We observed a null association between “revert to non-obese” with advanced fibrosis, with an RR of 0.70 (95% CI 0.14–3.54).

### 3.3. Assessment of the Public Health Impact of Weight Change in Populations

PAFs for population counterfactuals are reported in Table 4. In the weight loss scenario, if those who gained weight from early adulthood could have lost weight in later adulthood, 16.27% (95% CI 12.16–19.50%) of observed NAFLD cases in the total population might have been averted. In the weight maintenance scenario, if those who gained weight could have maintained non-obese during adulthood, 26.74% (95% CI 19.45–32.38%) of the incident NAFLD might have been prevented. In the partial prevention scenario, if the total population maintained non-obese (BMI < 30 kg/m^2^) during the life course, 26.67% (95% CI 18.17–33.23%) of NAFLD cases would have been prevented. In the comprehensive prevention scenario, keeping a normal weight (BMI < 25 kg/m^2^) across adulthood would have prevented 73.09% (95% CI 55.62–82.93%) of NAFLD cases. As for advanced fibrosis, if the total population had a normal BMI, 80.26% (95% CI 53.14–91.23%) of the case could have been averted (Table S4).

### 3.4. Sensitivity Analysis

Similar patterns of results were observed for mild fatty liver (Table S5) and moderate fatty liver (Table S6). The results were substantially unchanged when we removed participants with chronic diseases at baseline (Table S7). When evaluating the absolute weight changes (categorical variables), there was a dose–response association with risk of NAFLD (Table S8). Furthermore, we identified a J-shaped relationship between NAFLD and absolute weight change (continuous variables) during the three intervals (*p* for non-linear < 0.001, Figure S5A).

## 4. Discussion

Based on the nationally representative US adults’ cohort, we revealed the association between long-term weight change patterns across adulthood and NAFLD identified by VCTE among non-obese individuals. The lowest NAFLD risk was observed in stable non-obese individuals, whereas weight gain from early adulthood and middle and late adulthood were both at significantly elevated risk for NAFLD. Additionally, the risk of individuals who reverted to non-obese showed no significant difference compared with stable non-obese individuals. We further observed that a large percentage of NAFLD and advanced fibrosis cases would have been prevented with effective weight intervention in early adulthood.

We found strong evidence in support of the “risk raise” hypothesis. Those who gained weight to obese had an elevated risk of NAFLD. The association between absolute weight gain in midlife and NAFLD has been explicitly studied in previous longitudinal cohorts [11,25]. However, most studies were designed to assess the short-term effect of weight gain on incident NAFLD, and linear association was approximately reported. In this study, we reached a similar conclusion by using the long-term weight change through the adulthood as the exposure. In addition, several studies with the long-term track of weight have reported the linear relationship between the magnitude of weight growth and NAFLD risk [26,27], which were also seen in our current findings. In the study here, weight gain from a non-obese to an obese pattern from young to middle and middle to late adulthood had a 119% and 92% higher NAFLD risk, respectively. Moderate weight gain (ranging from 10.0 to 20.0 kg) and extreme weight gain (≥20.0 kg) from age 25 years to baseline were associated with a 166% and 279% higher NAFLD risk. Hence, our findings were generally consistent with previous studies.

We found that the effect of weight gain was generally stronger from young to middle adulthood than from middle to late adulthood. Previous studies have reported that weight gain from early adulthood is a risk factor for metabolic disorder [12,28], diabetes [17], hypertension [29], cardiovascular diseases [30]. Additionally, weight gain from earlier adulthood is more strongly associated with unfavorable metabolic indicators (e.g., adiponectin, C-peptide, HbA1c and gamma-glutamyl transferase) than weight gain from later adulthood [28,31]. Perennial accumulation of adipose tissue accumulation may result in abnormalities of free fatty acid [14]. Excessive free fatty acid transport to the liver and skeletal muscle leads to an increase in intrahepatic triglyceride and causes accumulation of liver fat [32,33]. Although the potential mechanism needs to be further studied, our results indicate that early prevention of weight gain would be an effective strategy to reduce future NAFLD risk.

Our findings were in support of the “risk elimination” hypothesis. In our study, participants who reverted to non-obese in midlife had a 52% lower risk of NAFLD when compared with the “early adulthood weight gain” group and even had a similar incidence rate with the “stable non-obese” group. The results were in line with previous prospective cohort and trial studies that weight loss through lifestyle interventions improve biomarkers of NAFLD [34,35]. A systematic review and meta-analysis also found a dose–response relationship between absolute weight loss and resolution of NAFLD [36]. In the current study, we estimated the PAF to explore the potential effect of weight loss intervention. As evaluated, a proportion of 16.27% NAFLD cases in the total population would be prevented if those who gained weight from early adulthood took measures to lose weight. We also found that 26.67% and 73.09% of NAFLD cases would be prevented if the total population maintained a non-obese or normal BMI throughout their life course, respectively. In total, a normal weight is associated with a greater improvement of NAFLD.

This study had several strengths. Taking advantage of NHANES, a large nationwide representative survey, our study had the unique feature of evaluating weight change across the life course and the development of NAFLD, and the results were more broadly generalizable to the US population. Our study shows the importance of weight change surveillance through an entire life because the underlying mechanisms of weight gain on NAFLD in certain life periods might differ. In early adulthood, weight gain was due in large part to accumulation of fat mass, whereas in later adulthoods, it was generally attributed to a decrease in lean mass [18]. Another strength of our research is the application of VCTE in fatty liver diagnosis, which further reduced information bias due to its accurate and quantitative features compared with abdomen ultrasound. Furthermore, we adjusted for adequate potential confounders to improve the stability of results.

Our study also had several limitations. First, we used recalled data on weight at age 25 and 10 years prior to baseline survey such that the misclassification bias could not be ignored. However, several validation studies suggested that self-reported weight was strongly correlated with anthropometric measures and could be used in life course epidemiology studies [37,38]. Second, we could not distinguish whether the weight change was intentional or unintentional, especially for those in the revert to non-obese group. Third, we did not capture the exact time of NAFLD onset, which may confound the effect of weight change on NAFLD.

## 5. Conclusions

In the non-obese individuals, weight gain across entire adulthood was independently associated with an increased risk of developing NAFLD. A larger reduction in NAFLD risk can be expected with losing weight in later life. Taken together, this study provides critical quantitative estimates that strategies for maintaining a healthy weight at the individual level may help reduce NAFLD progression and its associated consequences. At the national level, policies and programs developing to control the prevalence of obesity would be beneficial for the primary prevention of NAFLD.

## Figures and Tables

**Table 1:** Baseline characteristics of study participants in NHANES 2017–2018.

Characteristics		Weight Change Patterns				
	Total (*n* = 2212, 100%)	Stable Non-Obese (*n* = 1252, 56.13%)	Early Adulthood Weight Gain (*n* = 458, 21.44%)	Middle and Late Adulthood Weight Gain (*n* = 370, 17.33%)	Revert to Non-Obese (*n* = 132, 5.11%)	*p* Value
Age, years, mean ± SE	55.74 ± 0.47	55.24 ± 0.50	58.25 ± 0.90	52.58 ± 0.89	61.43 ± 1.30	<0.001
Sex, *n* (%)						0.009
Male	1056 (46.22)	611 (44.03)	223 (56.10)	148 (38.12)	74 (56.23)	
Female	1156 (53.78)	641 (55.97)	235 (43.90)	222 (61.88)	58 (43.77)	
BMI, kg/m^2^, mean ± SE						
At age 25 years	22.85 ± 0.12	21.77 ± 0.12	25.05 ± 0.31	23.56 ± 0.21	23.17 ± 0.36	<0.001
10 years prior	27.38 ± 0.21	24.20 ± 0.07	34.65 ± 0.30	27.10 ± 0.16	33.17 ± 0.66	<0.001
At baseline	29.11 ± 0.28	25.16 ± 0.14	35.95 ± 0.42	33.92 ± 0.16	27.47 ± 0.24	<0.001
Absolute weight change from age 25 years old to baseline, kg, mean ± SE	15.72 ± 0.56	7.95 ± 0.42	28.66 ± 0.95	26.68 ± 0.71	9.52 ± 1.42	<0.001
Waist circumference, cm, mean ± SE	100.32 ± 0.76	90.91 ± 0.40	117.87 ± 1.04	109.78 ± 0.65	98.13 ± 0.84	<0.001
Race and ethnicity, *n* (%)						<0.001
Non-Hispanic White	727 (64.00)	381 (61.52)	181 (71.55)	115 (61.73)	50 (67.34)	
Non-Hispanic Black	533 (11.03)	248 (9.24)	144 (12.39)	117 (15.93)	24 (8.42)	
Non-Hispanic Asian	385 (6.90)	330 (10.49)	14 (1.16)	32 (3.63)	9 (2.58)	
Hispanic	460 (13.54)	245 (14.29)	86 (9.14)	89 (15.41)	40 (17.44)	
Other	107 (4.53)	48 (4.46)	33 (5.76)	17 (3.30)	9 (4.23)	
Education, *n* (%)						0.158
Less than high school	416 (10.85)	230 (11.36)	81 (8.46)	68 (10.83)	37 (15.41)	
High school or equivalent	498 (25.63)	265 (23.55)	100 (24.34)	96 (32.51)	37 (30.64)	
College or above	1293 (63.52)	754 (65.10)	277 (67.19)	205 (56.66)	57 (53.95)	
Family income–poverty ratio level, *n* (%)						0.169
0–1.0	303 (10.06)	159 (9.21)	63 (7.71)	65 (15.04)	16 (11.88)	
1.1–3.0	838 (33.00)	472 (32.40)	183 (32.10)	132 (34.18)	51 (39.15)	
>3.0	806 (56.94)	480 (58.39)	157 (60.19)	130 (50.78)	39 (48.97)	
Marital status, *n* (%)						0.352
Married	1293 (64.95)	786 (67.45)	245 (62.35)	191 (61.68)	71 (59.47)	
Separated	581 (23.47)	286 (21.35)	154 (27.86)	103 (24.53)	38 (24.76)	
Never married	335 (11.58)	178 (11.20)	59 (9.80)	75 (13.78)	23 (15.77)	
Alcohol consumption, *n* (%)						0.005
Non-drinker	747 (27.84)	428 (29.48)	167 (27.98)	97 (18.39)	55 (41.72)	
Low to moderate drinker	1340 (72.16)	737 (70.52)	274 (72.02)	259 (81.61)	70 (58.28)	
Smoking status, *n* (%)						0.113
Never smoker	1284 (59.21)	752 (60.44)	242 (54.74)	216 (60.30)	74 (60.85)	
Former smoker	335 (12.71)	195 (13.05)	51 (9.45)	62 (13.19)	27 (20.90)	
Current smoker	593 (28.08)	305 (26.51)	165 (35.81)	92 (26.50)	31 (18.25)	
Leisure time physical activity level, *n* (%)						0.003
0 times/week	1199 (45.91)	633 (41.16)	275 (49.85)	207 (54.60)	84 (51.96)	
1–2 times/week	707 (38.06)	421 (38.55)	135 (40.34)	122 (36.03)	29 (29.90)	
≥3 times/week	304 (16.04)	196 (20.28)	48 (9.82)	41 (9.37)	19 (18.14)	
Healthy eating index score, *n* (%)						0.021
Quarter 1	381 (20.38)	174 (15.79)	106 (25.25)	76 (27.94)	25 (23.57)	
Quarter 2	484 (25.60)	257 (24.54)	115 (28.51)	79 (26.24)	33 (22.40)	
Quarter 3	514 (24.30)	291 (25.85)	107 (23.95)	92 (22.67)	24 (14.58)	
Quarter 4	667 (29.72)	422 (33.83)	108 (22.29)	99 (23.16)	38 (39.45)	
Chronic diseases, *n* (%)						
Diabetes	488 (15.31)	188 (8.254)	174 (32.15)	77 (13.46)	49 (28.35)	<0.001
Hypertension	1013 (39.03)	478 (30.92)	281 (52.56)	174 (42.63)	80 (57.13)	<0.001
Dyslipidemia	812 (34.13)	433 (31.06)	202 (44.02)	120 (30.09)	57 (39.15)	0.035

In total, 5, 265, 3, 125, 2, 166, 54 and 58 participants had missing information for baseline education, family income–poverty ratio, marital status, alcohol consumption, leisure time physical activity level, healthy eating index score, hypertension and dyslipidemia, respectively.

**Table 2:** Association of weight change patterns across adulthood with NAFLD in NHANES 2017–2018.

	Weight Change Patterns			
	Stable Non-Obese	Early Adulthood Weight Gain	Middle and Late Adulthood Weight Gain	Revert to Non-Obese
Number of NAFLD	355	298	226	45
Age adjusted incidence rate (%)	23.82 (19.66, 27.98)	67.20 (58.75, 75.64)	54.57 (46.82, 62.31)	37.40 (27.85, 46.95)
Model 1, RR (95% CI)	1.00 (ref)	2.77 (2.21, 3.49)	2.33 (1.78, 3.05)	1.37 (0.95, 1.98)
Model 2, RR (95% CI)	1.00 (ref)	2.23 (1.73, 2.88)	1.98 (1.45, 2.71)	1.09 (0.70, 1.68)
Model 3, RR (95% CI)	1.00 (ref)	2.19 (1.64, 2.91)	1.92 (1.40, 2.62)	1.01 (0.62, 1.64)

Model 1 was adjusted for age, sex, and race/ethnicity. Model 2 was additionally adjusted for education level, family income–poverty ratio level, marital status, alcohol consumption and smoking status, and chronic diseases. Model 3 was further adjusted for leisure time physical activity level and HEI. Incidence rates were directly standardized to age distribution of entire study population.

**Table 3:** Risk raise and risk elimination: RRs for weight change pattern and incident NAFLD.

Hypothesis	Weight Change Pattern	RR (95% CI)	*p* Value
Risk raise	Stable non-obese	1.00 (ref)	
	Early or middle and late adulthood weight gain	2.07 (1.60, 2.67)	<0.001
Risk elimination	Early adulthood weight gain	1.00 (ref)	
	Revert to non-obese	0.48 (0.37, 0.61)	<0.001

Risk estimates were adjusted for baseline age, sex, race/ethnicity, education level, family income–poverty ratio level, marital status, alcohol consumption and smoking status, chronic diseases, baseline leisure time physical activity level and HEI.

**Table 4:** PAFs for population counterfactuals of NAFLD.

Scenario	Definition	PAF (%), 95% CI of Non-Obese Population	PAF (%), 95% CI of Total Population
Weight loss	If those who gained weight from early adulthood could have lost weight in later adulthood (“early adulthood weight gain” group vs. “revert to non-obese” group).	18.98 (14.18, 22.74)	16.27 (12.16, 19.50)
Weight maintenance	If those who gained weight could have maintained non-obese during adulthood (“early or middle and late adulthood weight gain” group vs. “stable non-obese” group).	31.18 (22.69, 37.76)	26.74 (19.45, 32.38)
Partial prevention	If the total population maintained non-obese (BMI < 30 kg/m^2^) across adulthood (“stable non-obese” vs. other counterparts).	31.10 (21.19, 38.76)	26.67 (18.17, 33.23)
Comprehensive prevention	If the total population had a normal BMI (BMI < 25 kg/m^2^) across adulthood (“stable normal” vs. other counterparts).	73.32 (55.03, 82.05)	73.09 (55.62, 82.93)

Risk estimates were adjusted for baseline age, sex, race/ethnicity, education level, family income–poverty ratio level, marital status, alcohol consumption and smoking status, chronic diseases, baseline leisure time physical activity level and HEI.

## Data Availability

Detailed survey operation manuals, consent documents, and brochures are available on the NHANES website (https://www.cdc.gov/nchs/nhanes/about_nhanes, accessed on 9 May 2022).

## Supplementary Materials

The following supporting information can be downloaded at: https://www.mdpi.com/article/10.3390/nu14102140/s1. Figure S1. Flowchart for the selection of study participants; Figure S2. The relationship between weight change and CAP value; Figure S3. Associations between weight change patterns across adulthood and risk of NAFLD S3 stratified by age and sex; Figure S4. Associations between weight change patterns across adulthood and risk of NAFLD S3 stratified by race/ethnicity; Figure S5. Dose–response association between absolute weight change across adulthood and risk of NAFLD S3 and advanced fibrosis; Table S1. Pearson correlation coefficients for BMI at three time points and absolute weight change during three intervals; Table S2. Incidence rate ratios (95% confidence intervals) of NAFLD S3 with BMI at three time points in the NHANES 2017–2018; Table S3. Association between weight change patterns across adulthood and advanced fibrosis in NHANES 2017–2018; Table S4. Population attributable fractions (PAF) for population counterfactuals of advanced fibrosis; Table S5. Sensitivity analyses of the association between weight change patterns across adulthood and NAFLD S1 in NHANES 2017–2018; Table S6. Sensitivity analyses of the association between weight change patterns across adulthood and NAFLD S2 in NHANES 2017–2018; Table S7. Sensitivity analyses of the association between weight change patterns across adulthood and NAFLD S3 with exclusion of chronic diseases at baseline; Table S8. Association between absolute weight change across adulthood and NAFLD S3 in NHANES 2017–2018.
