# Supplementary material for: Weight Change across Adulthood in Relation to Non-Alcoholic Fatty Liver Disease among Non-Obese Individuals
Source: Nutrients. 2022 May 20;14(10):2140. doi: 10.3390/nu14102140 (PMC9144793; doi:10.3390/nu14102140)
Supplement: Supplementary file 1 [file nutrients-14-02140-s001.zip › nutrients-1721399-supplementary.pdf]

Contents

Figure S1. Flowchart for selection of study participants.

Figure S2. The relationship of weight change with CAP value.

Figure S3. Associations between weight change patterns across adulthood and risk of NAFLD S3 stratified by age and sex.

Figure S4. Associations between weight change patterns across adulthood and risk of NAFLD S3 stratified by race/ethnicity.

Figure S5. Dose-response association between absolute weight change across adulthood and risk of NAFLD S3 and advanced fibrosis.

Table S1. Pearson correlation coefficients for BMI at three time points and absolute weight change during three intervals.

Table S2. Incidence rate ratios (95% confidence intervals) of NAFLD S3 with BMI at three time points in the NHANES 2017-2018.

Table S3. Association between weight change patterns across adulthood and advanced fibrosis in NHANES 2017-2018.

Table S4. Population attributable fractions (PAF) for population counterfactuals of advanced fibrosis.

Table S5. Sensitivity analyses of the association between weight change patterns across adulthood and NAFLD S1 in NHANES 2017-2018.

Table S6. Sensitivity analyses of the association between weight change patterns across adulthood and NAFLD S2 in NHANES 2017-2018.

Table S7. Sensitivity analyses of the association between weight change patterns across adulthood and NAFLD S3 with exclusion of chronic diseases at baseline.

Table S8. Association between absolute weight change across adulthood and NAFLD S3 in NHANES 2017-2018.

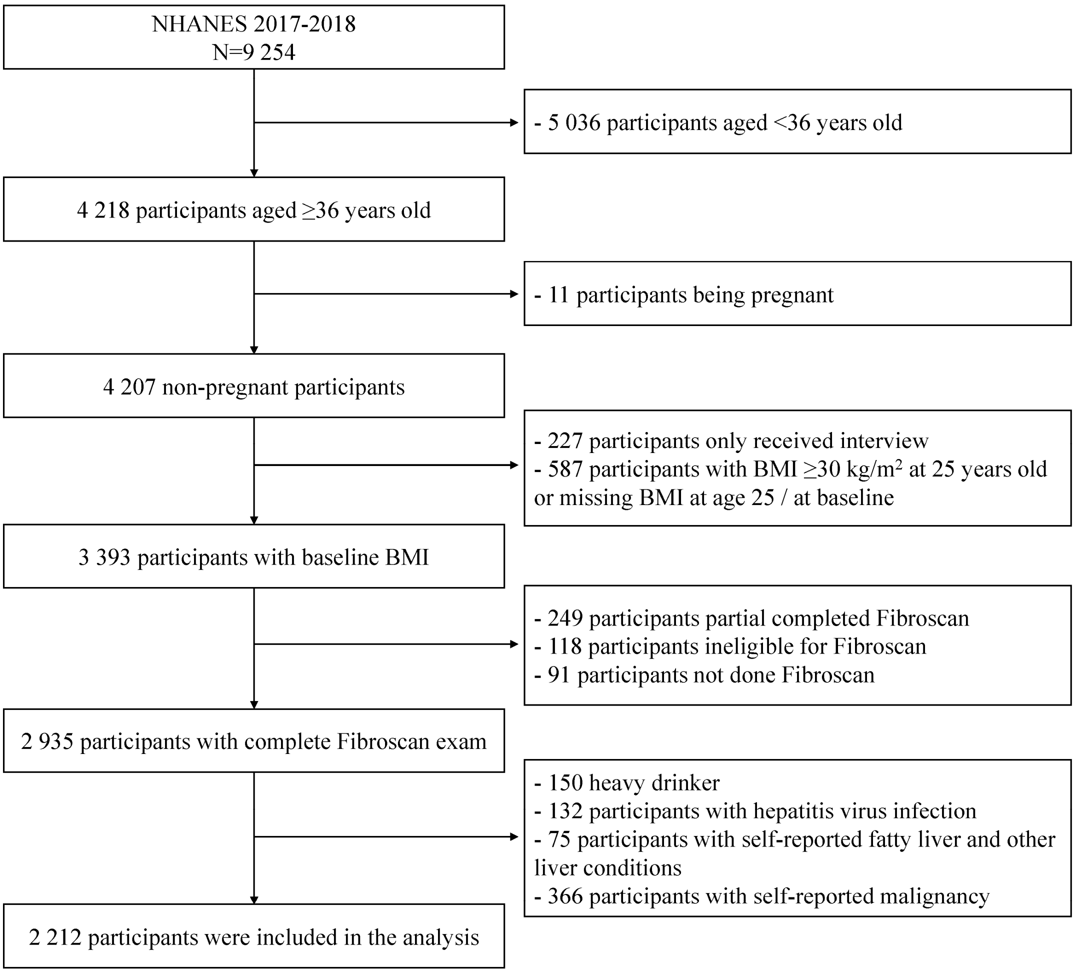

Figure S1. Flowchart for selection of study participants.

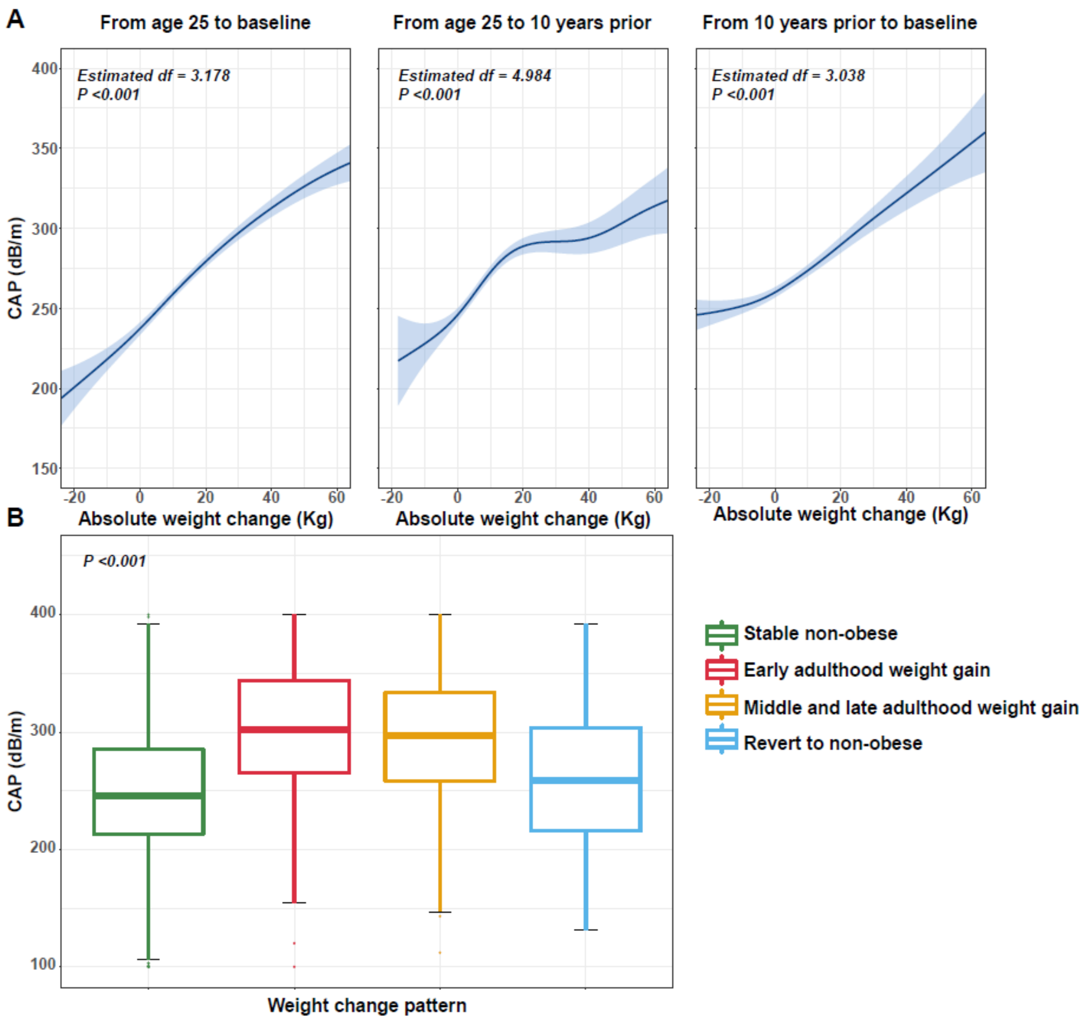

Figure S2. The relationship of weight change with CAP value. (**A**) The non-linear relations between absolute weight change and controlled attenuation parameter (CAP), assumed by a generalized additive model. (**B**) Change in CAP values by weight change patterns.

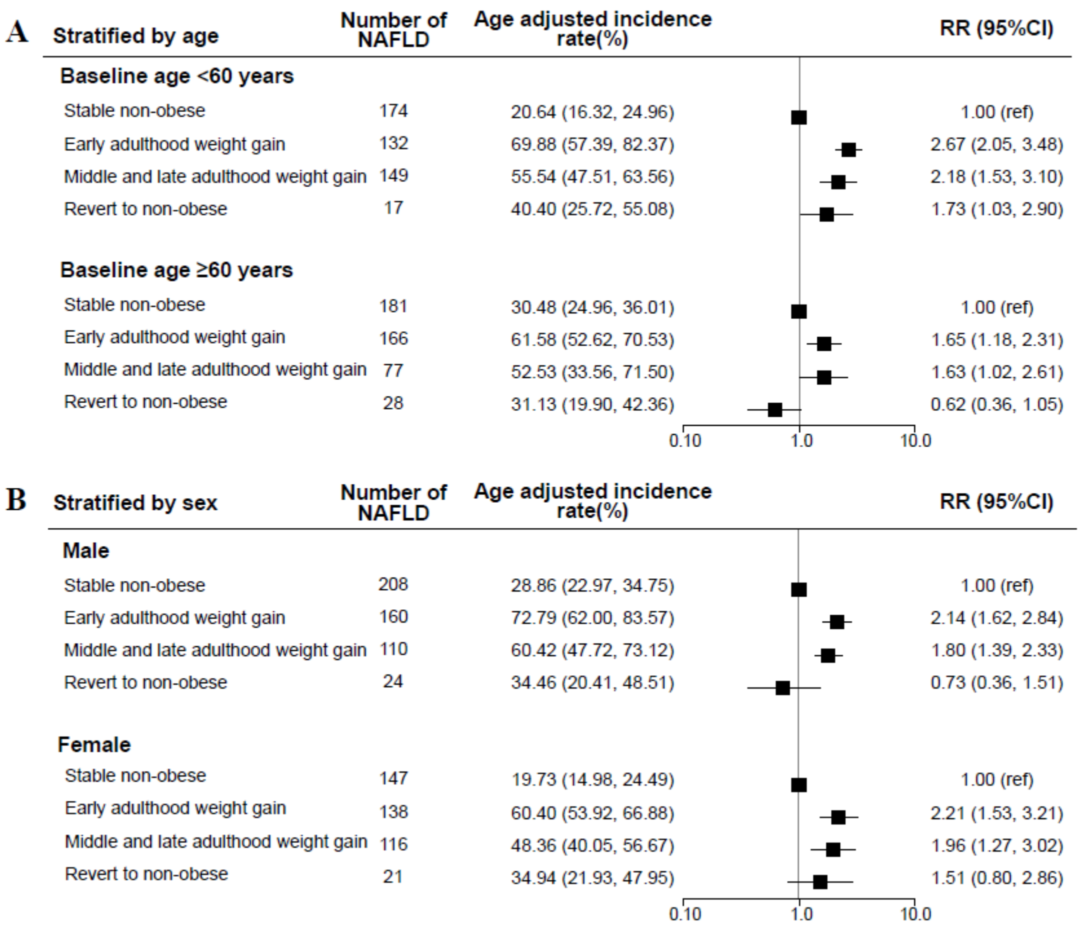

Figure S3. Associations between weight change patterns across adulthood and risk of NAFLD S3 stratified by age and sex. Risk estimates were adjusted for baseline age (**A**) (not adjusted in subgroup analysis by age), sex (**B**) (not adjusted in subgroup analysis by sex), race/ethnicity, education level, family income-poverty ratio level, marital status, alcohol consumption and smoking status, chronic diseases, baseline leisure time physical activity level and healthy eating index scores. In analysis of baseline age and sex, *P* for interaction were 0.015 and 0.231, respectively.

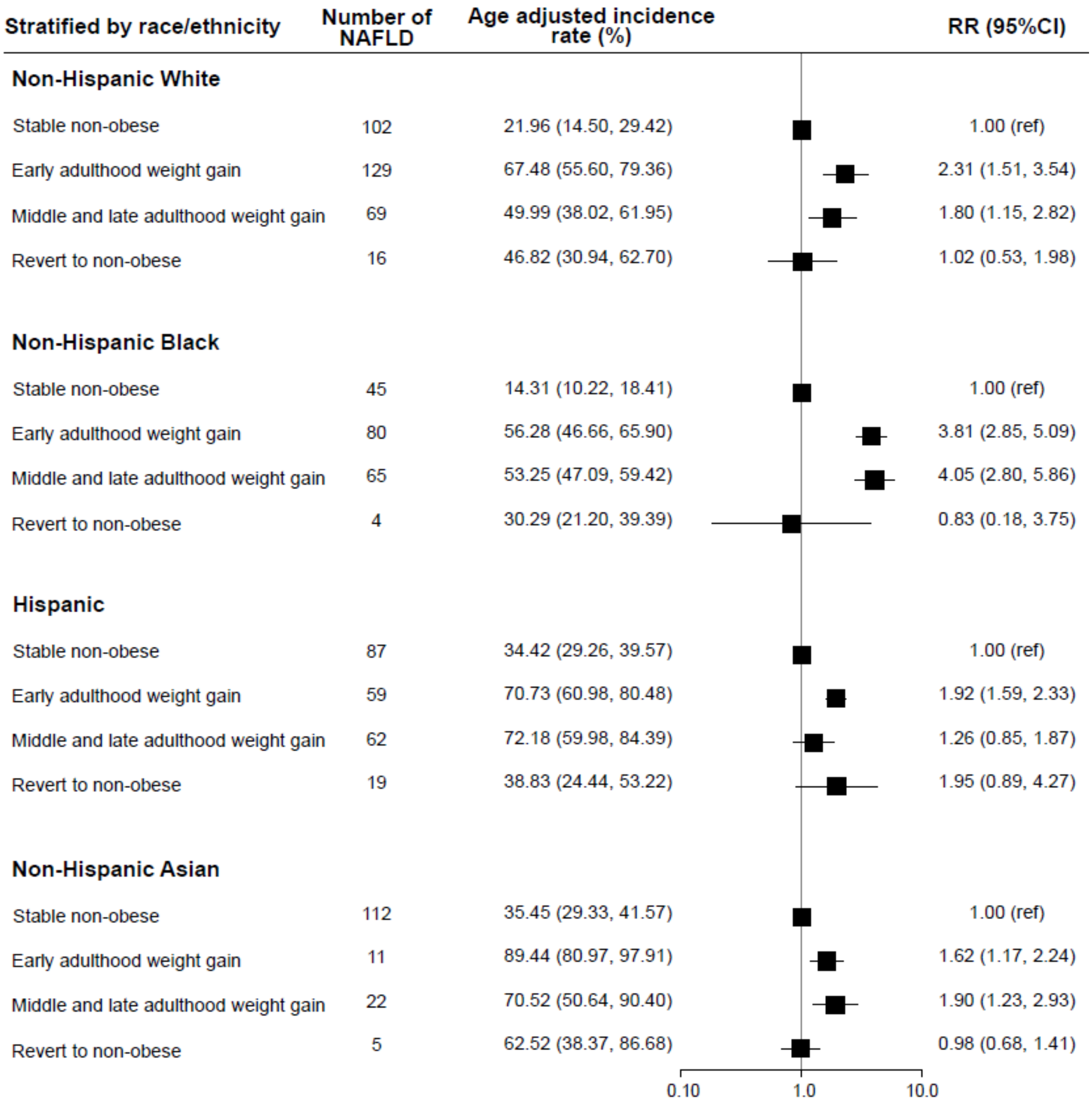

Figure S4. Associations between weight change patterns across adulthood and risk of NAFLD S3 stratified by race/ethnicity. Risk estimates were adjusted for baseline age, sex, education level, family income-poverty ratio level, marital status, alcohol consumption and smoking status, chronic diseases, baseline leisure time physical activity level and healthy eating index scores. Corresponding *P* for interaction was 0.844.

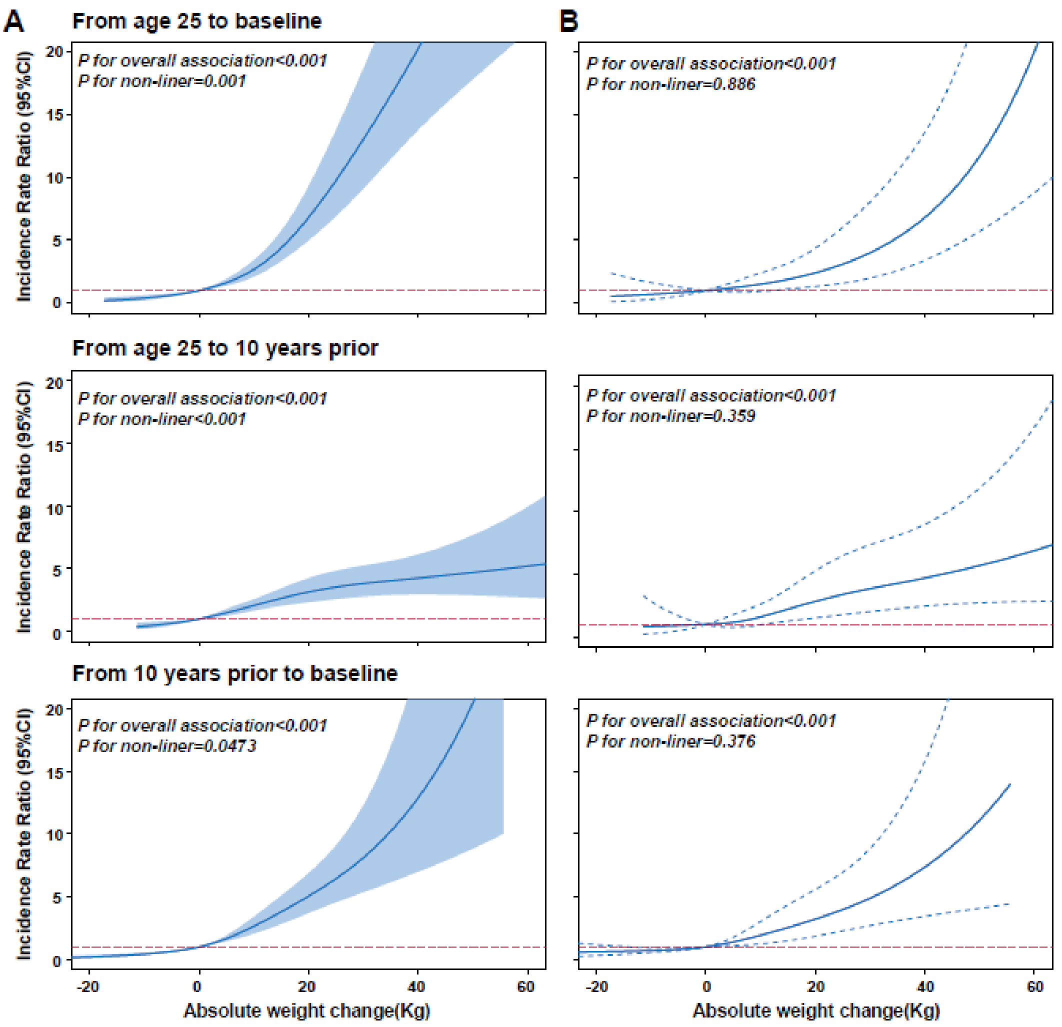

Figure S5. Dose-response association between absolute weight change across adulthood and risk of NAFLD S3 and advanced fibrosis. Associations were examined by multivariable linear regression models based on restricted cubic splines with 4 knots. All models were adjusted for baseline age, sex, race/ethnicity, education level, family income-poverty ratio level, marital status, alcohol consumption and smoking status, chronic diseases, baseline leisure time physical activity level and healthy eating index scores. For weight change from age 25 to baseline or 10 years prior, $BMI_{age\ 25}$ was also adjusted. For weight change from 10 years prior to baseline, $BMI_{10\ prior}$ was also adjusted. (**A**)

Association of absolute weight change with severe steatosis. Rate ratios are indicated by solid lines and 95% CIs by shaded areas. (**B**) Association of absolute weight change with advanced fibrosis. Rate ratios are indicated by solid lines and 95% CIs by dashed lines.

Table S1. Pearson correlation coefficients for BMI at three time points and absolute weight change during three intervals.

	BMI			Absolute Weight Change		
	At Age 25 Years	10 Years Prior	At Baseline	From Age 25 to 10 Years Prior	From Age 25 to Baseline	From 10 Years Prior to Baseline
**BMI at age 25 years**	1.000	-	-	-	-	-
**BMI 10 years prior**	0.491	1.000	-	-	-	-
**BMI at baseline**	0.397	0.668	1.000	-	-	-
**Weight change from age 25 to 10 years prior**	-0.088	0.793	0.490	1.000		
**Weight change from age 25 to baseline**	-0.099	0.409	0.845	0.577	1.000	-
**Weight change from 10 years prior to baseline**	-0.035	-0.272	0.528	-0.271	0.629	1.000

Table S2. Incidence rate ratios (95% confidence intervals) of NAFLD S3 with BMI at three time points in the NHANES 2017-2018.

	BMI Group (kg/m²)				
	**<18.5**	**18.5-24.9**	**25.0-29.9**	**30.0-34.9**	**≥35.0**
**BMI at age 25**					
Number of NAFLD	64	545	315	-	-
Age adjusted incidence rate (%)	32.65 (19.24, 46.06)	30.49 (26.77, 34.22)	56.71 (48.28, 65.13)	-	-
Model 1, RR (95% CI)	1.04 (0.73, 1.49)	1.00 (ref)	1.71 (1.36, 2.15)	-	-
Model 2, RR (95% CI)	1.14 (0.80, 1.61)	1.00 (ref)	1.54 (1.25, 1.90)	-	-
Model 3, RR (95% CI)	1.10 (0.77, 1.57)	1.00 (ref)	1.47 (1.21, 1.79)	-	-
**BMI at 10 years prior**					
Number of NAFLD	4	188	385	214	120
Age adjusted incidence rate (%)	15.87 (9.06, 22.68)	22.08 (16.48, 27.67)	41.87 (35.94, 47.80)	55.53 (45.97, 65.09)	65.78 (56.60, 74.97)
Model 1, RR (95% CI)	0.43 (0.20, 0.93)	1.00 (ref)	1.91 (1.40, 2.62)	2.61 (1.94, 3.50)	3.34 (2.43, 4.58)
Model 2, RR (95% CI)	0.55 (0.25, 1.19)	1.00 (ref)	1.84 (1.24, 2.73)	2.20 (1.58, 3.07)	2.64 (1.77, 3.95)
Model 3, RR (95% CI)	0.55 (0.24, 1.22)	1.00 (ref)	1.81 (1.23, 2.69)	2.16 (1.51, 3.07)	2.48 (1.62, 3.81)
**BMI at baseline**					
Number of NAFLD	1	79	320	270	254
Age adjusted incidence rate (%)	1.77 (-1.76, 5.29)	10.90 (5.73, 16.07)	34.60 (29.85, 39.35)	50.50 (42.26, 58.74)	76.55 (70.01, 83.10)
Model 1, RR (95% CI)	0.11 (0.01, 1.12)	1.00 (ref)	2.90 (1.82, 4.63)	4.32 (2.67, 6.98)	7.09 (4.26, 11.79)
Model 2, RR (95% CI)	0.14 (0.01, 1.32)	1.00 (ref)	3.07 (1.73, 5.44)	4.03 (2.23, 7.29)	6.22 (3.32, 11.65)
Model 3, RR (95% CI)	0.14 (0.01, 1.37)	1.00 (ref)	3.11 (1.74, 5.56)	4.11 (2.19, 7.72)	6.13 (3.20, 11.73)

All estimates accounted for complex survey designs. In analysis of BMI at 10 years prior to baseline, 24 participants were excluded due to missing values on $BMI_{10\ prior}$. Model 1 was adjusted for baseline age, sex, race/ethnicity. Model 2 was additionally adjusted for education level, family income-poverty ratio level, marital status, alcohol consumption and smoking status, and chronic diseases. Model 3 was further adjusted for baseline leisure time physical activity level and healthy eating index scores. Incidence rates were directly standardized to age distribution of entire study population.

Table S3. Association between weight change patterns across adulthood and advanced fibrosis in NHANES 2017-2018.

	Weight Change Patterns			
	Stable Non-Obese	Early Adulthood Weight Gain	Middle and Late Adulthood Weight Gain	Revert to Non-Obese
Number of fibrosis	31	66	23	5
Age adjusted incidence rate (%)	1.81 (0.28, 3.35)	11.73 (7.20, 16.25)	5.64 (3.11, 8.16)	1.48 (-0.13, 3.08)
Model 1, RR (95% CI)	1.00 (ref)	7.21 (3.02, 17.22)	4.44 (1.56, 12.60)	1.23 (0.29, 5.15)
Model 2, RR (95% CI)	1.00 (ref)	3.28 (1.26, 8.57)	2.64 (1.00, 6.98)	0.63 (0.13, 2.93)
Model 3, RR (95% CI)	1.00 (ref)	3.20 (1.19, 8.62)	2.46 (0.97, 6.20)	0.70 (0.14, 3.54)

All estimates accounted for complex survey designs. Model 1 was adjusted for baseline age, sex, race/ethnicity. Model 2 was additionally adjusted for education level, family income-poverty ratio level, marital status, alcohol consumption and smoking status, and chronic diseases. Model 3 was further adjusted for baseline leisure time physical activity level and healthy eating index scores. Incidence rates were directly standardized to age distribution of entire study population.

Table S4. Population attributable fractions (PAF) for population counterfactuals of advanced fibrosis.

Scenario	Definition	PAF (%), 95% CI of Non-Obese Population	PAF (%), 95% CI of Total Population
Weight loss	If those who gained weight from early adulthood instead loss weight during middle and late adulthood.	44.73 (25.10, 51.15)	35.66 (20.01, 40.77)
Weight maintenance	If those who gained weight during adulthood had not gained weight.	50.67 (9.97, 67.10)	40.39 (7.95, 53.48)
Partial prevention	If the total population maintained non-obese (BMI < 30 kg/m^2) during the life-course.	49.04 (0.42, 68.21)	39.09 (0.33, 54.37)
Comprehensive prevention	If the total population had a normal BMI (BMI < 25 kg/m^2) cross adulthood.	79.96 (52.94, 90.89)	80.26 (53.14, 91.23)

All estimates accounted for complex survey designs. Risk estimates were adjusted for baseline age, sex, race/ethnicity, education level, family income-poverty ratio level, marital status, alcohol consumption and smoking status, chronic diseases, baseline leisure time physical activity level and healthy eating index scores.

Table S5. Sensitivity analyses of the association between weight change patterns across adulthood and NAFLD S1 in NHANES 2017-2018.

	Weight Change Patterns			
	**Stable Non-Obese**	**Early Adulthood Weight Gain**	**Middle and Late Adulthood Weight Gain**	**Revert to Non-Obese**
Number of NAFLD	613	391	298	80
Age adjusted incidence rate (%)	43.76 (38.93, 48.59)	84.29 (76.45, 92.14)	76.25 (68.64, 83.85)	54.67 (43.89, 65.45)
Model 1, RR (95% CI)	1.00 (ref)	1.87 (1.57, 2.22)	1.73 (1.44, 2.08)	1.20 (0.98, 1.49)
Model 2, RR (95% CI)	1.00 (ref)	1.70 (1.41, 2.07)	1.64 (1.31, 2.06)	1.06 (0.81, 1.39)
Model 3, RR (95% CI)	1.00 (ref)	1.66 (1.37, 2.01)	1.58 (1.26, 1.99)	1.04 (0.77, 1.40)

All estimates accounted for complex survey designs. Model 1 was adjusted for baseline age, sex, race/ethnicity. Model 2 was additionally adjusted for education level, family income-poverty ratio level, marital status, alcohol consumption and smoking status, and chronic diseases. Model 3 was further adjusted for baseline leisure time physical activity level and healthy eating index scores. Incidence rates were directly standardized to age distribution of entire study population.

Table S6. Sensitivity analyses of the association between weight change patterns across adulthood and NAFLD S2 in NHANES 2017-2018.

	Weight Change Patterns			
	**Stable Non-Obese**	**Early Adulthood Weight Gain**	**Middle and Late Adulthood Weight Gain**	**Revert to Non-Obese**
Number of NAFLD	454	333	253	59
Age adjusted incidence rate (%)	30.70 (25.86, 35.54)	75.18 (67.81, 82.56)	65.13 (54.48, 75.78)	44.39 (32.78, 56.00)
Model 1, RR (95% CI)	1.00 (ref)	2.42 (1.89, 3.09)	2.12 (1.59, 2.82)	1.32 (1.02, 1.72)
Model 2, RR (95% CI)	1.00 (ref)	2.04 (1.54, 2.69)	1.87 (1.37, 2.55)	1.11 (0.78, 1.59)
Model 3, RR (95% CI)	1.00 (ref)	1.96 (1.46, 2.62)	1.77 (1.30, 2.41)	1.06 (0.72, 1.58)

All estimates accounted for complex survey designs. Model 1 was adjusted for baseline age, sex, race/ethnicity. Model 2 was additionally adjusted for education level, family income-poverty ratio level, marital status, alcohol consumption and smoking status, and chronic diseases. Model 3 was further adjusted for baseline leisure time physical activity level and healthy eating index scores. Incidence rates were directly standardized to age distribution of entire study population.

Table S7. Sensitivity analyses of the association between weight change patterns across adulthood and NAFLD S3 with exclusion of chronic diseases at baseline.

	Weight Change Patterns			
	**Stable Non-Obese**	**Early Adulthood Weight Gain**	**Middle and Late Adulthood Weight Gain**	**Revert to Non-Obese**
Number of NAFLD	111	61	74	3
Age adjusted incidence rate (%)	20.87 (14.53, 27.20)	55.27 (42.26, 68.27)	41.33 (33.47, 49.18)	9.06 (-0.92, 19.03)
Model 1, RR (95% CI)	1.00 (ref)	3.00 (2.06, 4.37)	2.34 (1.66, 3.31)	0.93 (0.26, 3.41)
Model 2, RR (95% CI)	1.00 (ref)	2.92 (1.88, 4.54)	2.25 (1.38, 3.69)	1.08 (0.29, 4.11)
Model 3, RR (95% CI)	1.00 (ref)	2.76 (1.89, 4.02)	2.10 (1.35, 3.27)	0.74 (0.12, 4.70)

All estimates accounted for complex survey designs. Model 1 was adjusted for baseline age, sex, race/ethnicity. Model 2 was additionally adjusted for education level, family income-poverty ratio level, marital status, alcohol consumption and smoking status. Model 3 was further adjusted for baseline leisure time physical activity level and healthy eating index scores. Incidence rates were directly standardized to age distribution of entire study population.

Table S8. Association between absolute weight change across adulthood and NAFLD S3 in NHANES 2017-2018.

	Absolute Weight Change				
	**Weight Change within 2.5 kg**	**Weight Loss of at Least 2.5 kg**	**Weight Gain of at Least 2.5 kg but Less than 10.0 kg**	**Weight Gain of at Least 10 kg but Less than 20.0 kg**	**Weight Gain of at Least 20.0 kg**
**From age 25 to baseline**					
Number of NAFLD	66	61	247	411	597
Age adjusted incidence rate (%)	22.27 (14.25, 30.29)	27.78 (17.84, 37.71)	45.89 (36.97, 54.81)	60.97 (55.51, 66.44)	82.23 (77.53, 86.94)
Model 1, RR (95% CI)	1.00 (ref)	1.44 (0.79, 2.62)	2.09 (1.58, 2.78)	2.81 (2.07, 3.82)	3.88 (3.03, 4.98)
Model 2, RR (95% CI)	1.00 (ref)	1.42 (0.75, 2.69)	2.12 (1.53, 2.94)	2.59 (1.84, 3.64)	3.70 (2.83, 4.84)
Model 3, RR (95% CI)	1.00 (ref)	1.37 (0.72, 2.61)	2.13 (1.53, 2.95)	2.66 (1.90, 3.73)	3.79 (2.88, 4.98)
**From age 25 to 10 years prior**					
Number of NAFLD	245	34	382	379	326
Age adjusted incidence rate (%)	44.50 (38.54, 50.46)	37.77 (26.96, 48.58)	55.92 (47.59, 64.25)	75.94 (69.60, 82.29)	73.24 (64.13, 82.35)
Model 1, RR (95% CI)	1.00 (ref)	0.94 (0.60, 1.49)	1.25 (0.96, 1.63)	1.67 (1.38, 2.02)	1.84 (1.57, 2.15)
Model 2, RR (95% CI)	1.00 (ref)	0.96 (0.59, 1.55)	1.10 (0.83, 1.45)	1.47 (1.18, 1.83)	1.59 (1.32, 1.92)
Model 3, RR (95% CI)	1.00 (ref)	0.91 (0.55, 1.52)	1.08 (0.82, 1.43)	1.48 (1.18, 1.85)	1.59 (1.31, 1.94)
**From 10 years prior to baseline**					
Number of NAFLD	222	303	402	251	188
Age adjusted incidence rate (%)	44.59 (39.36, 49.83)	45.28 (35.02, 55.54)	56.80 (51.94, 61.67)	67.03 (58.08, 75.99)	89.87 (83.65, 96.09)
Model 1, RR (95% CI)	1.00 (ref)	0.99 (0.78, 1.26)	1.23 (1.07, 1.42)	1.44 (1.14, 1.82)	1.96 (1.65, 2.31)
Model 2, RR (95% CI)	1.00 (ref)	0.92 (0.71, 1.20)	1.19 (1.02, 1.39)	1.36 (1.08, 1.70)	1.77 (1.45, 2.17)
Model 3, RR (95% CI)	1.00 (ref)	0.79 (0.64, 0.99)	1.18 (1.02, 1.38)	1.35 (1.09, 1.68)	1.70 (1.39, 2.08)

All estimates accounted for complex survey designs. Model 1 was adjusted for baseline age, sex, race/ethnicity. Model 2 was additionally adjusted for education level, family income-poverty ratio level, marital status, alcohol consumption and smoking status, and chronic diseases. Model 3 was further adjusted for baseline leisure time physical activity level and healthy eating index scores. Incidence rates were directly standardized to age distribution of entire study population.
